# Differential Response of Hippocampal and Cerebrocortical Autophagy and Ketone Body Metabolism to the Ketogenic Diet

**DOI:** 10.3389/fncel.2021.733607

**Published:** 2021-08-11

**Authors:** Daniela Liśkiewicz, Arkadiusz Liśkiewicz, Marta M. Nowacka-Chmielewska, Mateusz Grabowski, Natalia Pondel, Konstancja Grabowska, Sebastian Student, Jaroslaw J. Barski, Andrzej Małecki

**Affiliations:** ^1^Laboratory of Molecular Biology, Institute of Physiotherapy and Health Sciences, The Jerzy Kukuczka Academy of Physical Education, Katowice, Poland; ^2^Department of Physiology, Faculty of Medical Sciences in Katowice, Medical University of Silesia, Katowice, Poland; ^3^Department for Experimental Medicine, Faculty of Medical Sciences in Katowice, Medical University of Silesia, Katowice, Poland; ^4^Institute of Automatic Control, Silesian University of Technology, Gliwice, Poland; ^5^Biotechnology Centre, Silesian University of Technology, Gliwice, Poland

**Keywords:** ketogenic diet, acetoacetate, autophagy, ketolysis, ketogenesis, ketosis, β-hydroxybutyrate

## Abstract

Experimental and clinical data support the neuroprotective properties of the ketogenic diet and ketone bodies, but there is still a lot to discover to comprehensively understand the underlying mechanisms. Autophagy is a key mechanism for maintaining cell homeostasis, and therefore its proper function is necessary for preventing accelerated brain aging and neurodegeneration. Due to many potential interconnections, it is possible that the stimulation of autophagy may be one of the mediators of the neuroprotection afforded by the ketogenic diet. Recent studies point to possible interconnections between ketone body metabolism and autophagy. It has been shown that autophagy is essential for hepatic and renal ketogenesis in starvation. On the other hand, exogenous ketone bodies modulate autophagy both *in vitro* and *in vivo*. Many regional differences occur between brain structures which concern i.e., metabolic responses and autophagy dynamics. The aim of the present study was to evaluate the influence of the ketogenic diet on autophagic markers and the ketone body utilizing and transporting proteins in the hippocampus and frontal cortex. C57BL/6N male mice were fed with two ketogenic chows composed of fat of either animal or plant origins for 4 weeks. Markers of autophagosome formation as well as proteins associated with ketolysis (BDH1—3-hydroxybutyrate dehydrogenase 1, SCOT/OXCT1—succinyl CoA:3-oxoacid CoA transferase), ketone transport (MCT1—monocarboxylate transporter 1) and ketogenesis (HMGCL, HMGCS2) were measured. The hippocampus showed a robust response to nutritional ketosis in both changes in the markers of autophagy as well as the levels of ketone body utilizing and transporting proteins, which was also accompanied by increased concentrations of ketone bodies in this brain structure, while subtle changes were observed in the frontal cortex. The magnitude of the effects was dependent on the type of ketogenic diet used, suggesting that plant fats may exert a more profound effect on the orchestrated upregulation of autophagy and ketone body metabolism markers. The study provides a foundation for a deeper understanding of the possible interconnections between autophagy and the neuroprotective efficacy of nutritional ketosis.

## Introduction

The ketogenic diet is a very-low-carbohydrate, high-fat nutritional approach that induces a metabolic shift to the use of ketone bodies as a significant energy source. For decades, the ketogenic diet has been employed to manage drug-resistant epilepsy (Gano et al., 2014), but recently it is increasingly considered as an alternative or add-on therapy in many other pathological conditions, including central nervous system (CNS) disorders (Włodarek, 2019). The available data suggests that besides its well-known metabolic effects, including ketonemia, and decreased blood glucose and insulin levels, the ketogenic diet has many other beneficial health effects (Maalouf et al., 2009). There is evidence that the ketogenic diet improves cellular metabolism and mitochondrial biogenesis and reduces oxidative stress (Paoli et al., 2013). A large number of studies have demonstrated the neuroprotective properties of the ketogenic diet (reviewed in Yang et al., 2019), but there is still a lot to discover to comprehensively understand the underlying mechanisms. The ketogenic diet results in a number of changes observed in the brain on a molecular and cellular level, including an improvement of the mitochondrial function and biogenesis, modulation of the ATP-sensitive potassium channel, enhancement of purinergic and GABAergic neurotransmission, as well as antioxidant and anti-inflammatory effects (Gasior et al., 2006; Maalouf et al., 2009; Masino and Rho, 2012). It has been proposed that the stimulation of autophagy may be one of the mediators of the neuroprotection afforded by the ketogenic diet (McCarty et al., 2015). Autophagy as a cellular recycling mechanism is essential for the maintenance of cell homeostasis and viability, especially during stress conditions (Gerónimo-Olvera and Massieu, 2019). The impairment of autophagy results in cellular dysfunction and abnormal protein accumulation, leading to degenerative changes in mammalian tissues and contributing to the onset of many pathologies, including neurodegeneration (reviewed in Nah et al., 2015). Autophagy is induced in response to increased cellular energetic needs caused e.g., by nutrient deprivation or physical exercise, which are known autophagy inducers not only in peripheral tissues (He et al., 2012a; Bagherniya et al., 2018), but also, in the brain (Alirezaei et al., 2010; He et al., 2012b). The ketogenic diet mimics the biochemical actions of fasting and exerts many physiological and cellular responses similar to those evoked by intermittent energy restriction arising from food deprivation or exercise. The ketogenic diet may induce autophagy through at least a couple of mechanisms; in spite of this, the relationship between nutritional ketosis and autophagy stays largely unexplored. Recently, we have shown that 4-weeks-long feeding with the ketogenic diet caused the upregulation of hepatic autophagy, and the effect was likely dependent on the diet composition (Liśkiewicz et al., 2021). In the present study, we aimed to evaluate the influence of the ketogenic diet on brain autophagy. Here, we report that treatment with the ketogenic diet causes the upregulation of the numbers of autophagic structures in the mouse hippocampus and cerebral cortex, but the hippocampus tends to show a stronger response. Subsequently, we assumed that regional differences in response to the ketogenic diet may be associated with differences in the metabolic profiles of the hippocampus and cerebral cortex. It is known that different brain regions show significant differences in fuel metabolism, including glucose (Kleinridders et al., 2018) and fatty acids (Lee et al., 2013). Scarce data are available in regard to regional differences in ketone body metabolism in the CNS. Brain ketone body metabolism is determined by blood ketone body concentration, transport across the blood-brain barrier (BBB) and into cells, and the activity of ketone body metabolizing enzymes. BBB permeability to ketone bodies increases in response to prolonged ketonemia both in humans and rodents (Leino et al., 2001). Regional differences in BHB (β-hydroxybutyrate) uptake and MCT1 distribution have been also observed in the adult rodent brain (Bilger and Nehlig, 1992; Pellerin et al., 1998). However, there is no data showing regional adaptations of ketone body metabolism regulating enzymes to prolonged nutritional ketosis induced by the ketogenic diet.

Here, we investigated the local concentrations of ketone bodies BHB and AcAc (acetoacetate), the levels of MCT1 responsible for BHB transport to the brain, and the levels of ketolytic enzymes: SCOT/OXCT1 and BDH1. In comparison to the frontal cortex, the hippocampus showed a stronger response to nutritional ketosis in both changes in the markers of autophagy as well as levels of ketone body utilizing and transporting proteins, which was also accompanied by increased concentrations of ketone bodies in this brain structure.

## Materials and Methods

### Animals and Diets

Male C57BL/6 mice with an age of 9–10 weeks were provided by the Animal House of the Department for Experimental Medicine, Medical University of Silesia (Katowice, Poland) and were treated in accordance with the 2010/63/EU directive for animal experiments using the protocols approved and monitored by the Local Committee for Animal Experiments. The minimum number of mice required to obtain consistent data were used, and every effort was taken to minimize the suffering of the animals.

Three different chows were used in the study: standard rodent chow (control diet), ketogenic chow with a higher content of animal-based fats (chow A), and ketogenic chow enriched with fats of plant origin (chow P). All chows were prepared by the local supplier “Wytwórnia Pasz Morawski” (Kcynia, Poland). The composition and nutritional profile of the diets is provided in Supplementary Tables 1, 2, additionally the detailed FA (Fatty Acids) content of the diets was described previously (Liśkiewicz et al., 2021).

### Experimental Design

In the experiment 1 (exp. 1), thirty mice were divided into three groups (*n* = 10 in each group). Animals in the KA and KP group were fed with two different ketogenic chows A and P, respectively, for 4 weeks. In the control group (SD), the animals were maintained on the standard rodent chow. Animal weight was monitored weekly and blood ketosis was determined on the last day of the experiment (Supplementary Figure 1). The food was changed and weighted three times per week. Brain specimens collected from half of the animals from each group were used for microscopic analysis, and the other half were used to obtain cerebrocortical and hippocampal tissue homogenates for western blotting (brain regions from one hemisphere) and RNA samples for qRT-PCR (tissue collected from the other hemisphere). An additional experiment (exp. 2) was performed in order to evaluate the short-term influence of the ketogenic diet on autophagic markers. Twenty-four mice were divided into six groups (*n* = 4 in each group). In four groups, animals were fed with ketogenic diets A and P for 24 (KA24, KP24) or 48 h (KA48, KP48), respectively. In the F24 group animals were deprived of food for 24 h and in the control group (SD) animals were fed with the standard chow. Animal blood ketosis was measured daily (Supplementary Figure 2). Cerebrocortical and hippocampal samples were collected and further analyzed by immunoblotting.

### Sample Collection

In order to collect samples for RNA and protein isolation, the animals were decapitated. Afterward, the brain was rapidly removed and put on an ice-chilled metal plate, ventral side up. The entire cerebellum and brainstem were removed. The brain was turned dorsal side up and hemisected. After the removal of the hypothalamus, septum, and striatum, the hippocampi were removed using a curved forceps. Subsequently, a 2.0 mm-thick slice of the prefrontal cortex (PFC) was cut off from each hemisphere. The brain structures were immersed in RNAlater^®^ (Sigma-Aldrich Corp.) or immediately frozen on dry ice (for future preparation of cell lysates) and stored at –80°C until usage.

In order to collect brain specimens for microscopic analysis, the mice were anesthetized (i.p. injection of 100 mg/kg ketamine plus 10 mg/kg xylazine) and sacrificed by a transcardiac perfusion with Tris-Buffered Saline (TBS, pH 7.4, 4°C) followed by a fixative buffer of 10% formalin in TBS (pH 7.4, 4°C). The brains were removed and immersed in a fixative buffer for 24 h. The buffer was replaced with TBS + 0.02 sodium azide and the tissue was stored until processing.

### Measurement of Ketone Bodies

The blood BHB measurements were performed with a glucometer (Optium Xido Neo, Abbott Laboratories) in a drop of whole blood collected from the tip of the tail.

Brain levels of BHB were measured in tissue homogenates with Beta-Hydroxybutyric Acid ELISA Kit (Abbexa Ltd., abx514704) according to the manufacturer’s instruction. An equal volume of each sample was taken for analysis and the BHB concentration was normalized to the amount of protein in the sample.

AcAc concentration was measured in the cortex and hippocampus collected for 24 h before the assay (Acetoacetate Colorimetric Assay Kit, Sigma Aldrich, MAK199) and stored at –80°C, which allowed the avoidance of its biodegradation (Puchalska et al., 2021). The tissue was homogenized in MilliQ ultrapure water (100 μl per 10 mg of tissue), centrifuged, and used for the assay. Results were expressed as a mean ± standard deviation (SD).

### Western Blotting

For protein extraction, thawed tissue pieces were homogenized by sonication in RIPA buffer (500 μl for each 10 mg of tissue; Sigma-Aldrich Corp.) with protease (cOmplete^TM^ ULTRA Tablets, Roche Holding AG) and phosphatase (PhosSTOP^TM^, Roche Holding AG) inhibitors and then centrifuged at 12,000 x g for 20 min at 4°C. Protein concentration was determined with Roti^®^-Quant Universal (Carl Roth GmbH + Co., KG).

The samples containing 20 μg of total protein were separated on 4–15% or 8–16% (to separate two forms of LC3 protein) SDS- PAGE precast gels (Bio-Rad Laboratories) and transferred onto PVDF membranes (Bio-Rad Laboratories, Inc.). The membranes were blocked for 2 h at room temperature in Casein Blocking Buffer (Sigma-Aldrich Corp.) and incubated overnight at 4°C in the same blocking buffer with the following antibodies: Rabbit anti-MCT1 (1:1,000; Abcam, ab93048), rabbit anti-BDH1 (1:1,000; Sigma-Aldrich Corp., HPA030947), rabbit anti-OXCT1/SCOT (1:1,000; Sigma-Aldrich Corp., HPA012047), rabbit anti-LC3B antibody (1:500; Sigma-Aldrich Corp., L7543), rabbit anti-p62/SQSTM1 antibody (1:750; Sigma-Aldrich Corp., P0067), rabbit anti-Beclin-1 antibody (1:500; Sigma-Aldrich Corp., SAB1306484), or rabbit anti-β-actin (1:5,000; Sigma-Aldrich Corp.). After washing, the membranes were incubated with a secondary goat anti-rabbit IgG antibody (1:5,000, Invitrogen). Immunoblots were visualized by Clarity Western ECL Blotting Substrates (Bio-Rad Laboratories Inc.) and detected with the ChemiDoc^TM^ Touch Imaging System (Bio-Rad Laboratories Inc.). Targeted proteins were quantified using ImageLab Software 5.2.1 (Bio-Rad Laboratories Inc.). After normalization to the loading control (β-actin), the results were expressed as a mean ± SD fold change of the test samples in comparison with the control.

### RNA Extraction and qRT-PCR

Total RNA was extracted using the Ribospin^TM^ Kit (Geneall Biotechnology Co., Ltd.) in accordance with the manufacturer’s protocol. RNA samples were qualitatively and quantitatively evaluated by spectrophotometry (BioPhotometer, Eppendorf AG). For genomic DNA elimination, RNA samples were incubated with double strand-specific DNase for 2 min at 37°C. Reverse transcription was performed with the Transcriptor First Strand cDNA Synthesis Kit (Roche Holding AG) according to the manufacturer’s instruction using 100 ng total RNA and random hexamer primers. Samples were stored at –80°C.

The qRT-PCR analysis of mRNA expression levels was performed on a LightCycler96 Instrument (Roche Holding AG) and FastStart Essential DNA Green Master (Roche Holding AG). Primer-BLAST software (Ye et al., 2012) was used to design primers targeting a 111 bp sequence of LC3B (input sequence: NM_001364358.1; primers: 5′-GGGACCCTAACCCCATAGGA-3′ and 5′-TCTCCCCCTTGTATCGCTCT-3′), a 120 bp sequence of LC3A (input sequence: NM_025735.3 primers: 3′-TCCCCAGTGGATTAGGCAGA-5′ and 3′-ACCCAAAAGAGCAACCCGAA-5′) and a 98 bp fragment of p62/SQSTM1 mRNA (input sequence: NM_001290769; primers: 5′-AGGAACAGATGGAGTCGGGA-3′ and 5′-CCGGGGATCAGCCTCTGTAG-3′). PCR cycling conditions were 10 min at 95°C, 38 cycles of three-step amplification (10 s at 95°C, 10 s at 55°C and 10 s at 72°C) and melting curve analysis (10 s at 95°C, 1 min at 65°C and 1 s at 97°C). All samples were run in duplicate. An analysis of the melting curve verified the specificity of the PCR products. The relative quantification of the studied genes was performed with normalized to β-actin mRNA levels (input sequence NM_007393.5; primers: 5′-CTGTCGAGTCGCGTCCA-3′, 5′-TCCATGGCGAACTGGTGG-3′). The data was calculated using the 2^–ΔΔ*Ct*^ method (Livak and Schmittgen, 2001). The relative mRNA levels were expressed as a mean ± SD fold change of the test samples vs. the calibrator.

### Immunofluorescence, Confocal Microscopy, and Image Analysis

The fixed brains were dehydratated in increasing gradients of sacharose solutions (10, 20, and 30%) and embedded in OCT Compound, sectioned coronally (25-μm thick) on cryostat, and stored in 50% glycerol in TBS at –20°C. The floating slices were permeabilized (0.1% Triton x-100, RT, 1 h), blocked (5% normal donkey serum, Abcam, ab7475; 4°C, overnight), and incubated with primary rabbit anti-LC3 antibody (1:400, Sigma-Aldrich Corp., L7543; 4°C, 48 h) following donkey anti-rabbit Alexa Fluor 568 secondary antibody (1:500, Abcam, ab175694; 4°C, overnight). Afterward, slices were mounted onto glass microscope slides with Glycerol Mounting Medium with DAPI and DABCO^TM^ (Abcam, ab188804). Images of the hippocampus and cortex were acquired using a confocal microscope (Olympus FluoView FV 1000) with z-series followed by counting the LC3-positive puncta using Image J software (Fiji Distribution, NIH 22743772). After background subtraction, a 3D object counter plug-in with automatic thresholding was used to count objects of a volume between 6 and 250 voxels, which corresponds to puncta with a radius between 300 nm and 1 μm. The data was normalized to the volume of the slice and expressed as mean ± SD.

### Statistical Analysis

Statistical analysis was performed using GraphPad Prism 9.0.0 software (GraphPad Software Inc., United States). The distribution of each data set was checked for normality using the Shapiro–Wilk test. Normally distributed data were analyzed with the one-way ANOVA, followed by the Tukey *post hoc* test. Data with a non-normal distribution were analyzed with the Kruskal–Wallis test with Dunn’s *post hoc*. In all analyses, *p*-values of less than 0.05 were considered to be statistically significant.

## Results

In order to evaluate the influence of the ketogenic diet on autophagy and ketone body metabolism in different regions of the brain, markers of these processes were measured in the hippocampus and cerebral cortex of animals fed with two different ketogenic chows for 4 weeks. Animals fed with ketogenic diets have a substantial level of nutritional ketosis (average: 3.1 + 1.01 mM in the KA group and 3.4 + 0.54 mM in the KP group, Supplementary Figure 1A). Animals’ weight was stable in all groups throughout the course of the experiment, except an initial drop in the first week of the experiment in the KP group (please see the details in Supplementary Figure 1B).

### Autophagy Response to the Ketogenic Diet Depends on the Brain Region and Diet Composition

#### Hippocampal Formation of Autophagosomes Was Enhanced in Response to Ketogenic Diets

**FIGURE 1:**
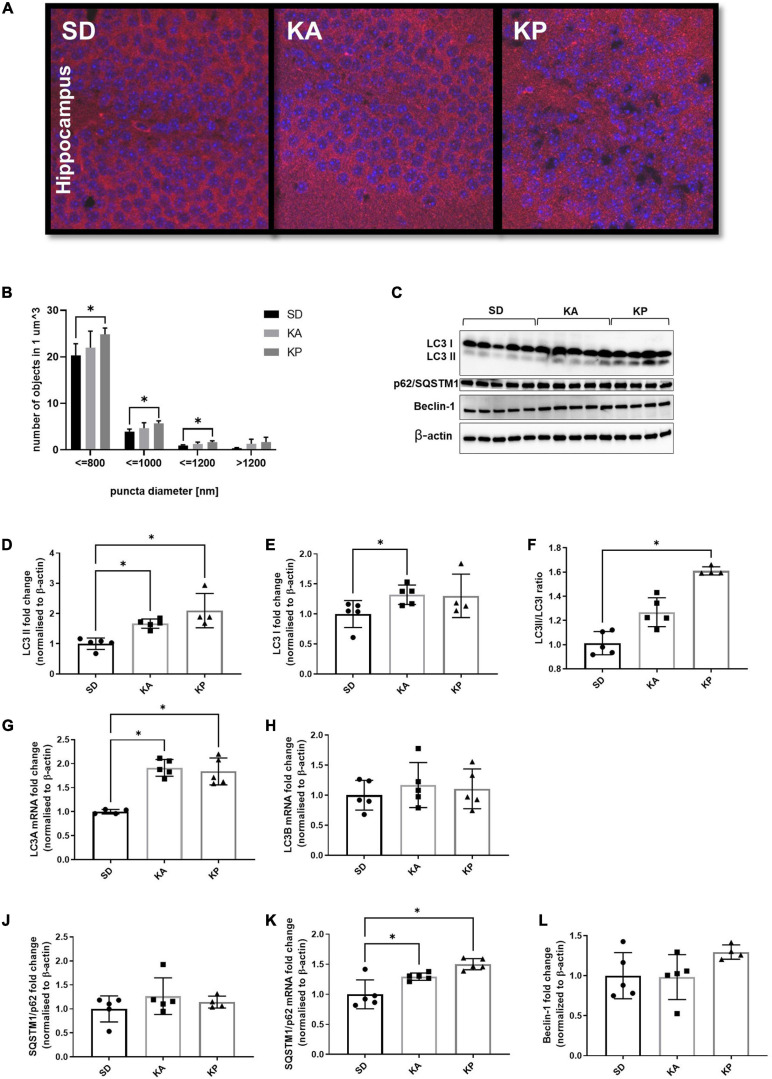
The level of autophagy markers in the hippocampus of the mice fed with the ketogenic diets. **(A)** The hippocampi were immunolabeled against LC3 protein (red) and the level of LC3-positive puncta was counted in dentate gyrus (*n* = 5). The nuclei are shown in blue (DAPI). **(B)** Number of LC3 puncta per 1 μm^3 of tissue; the counted objects were divided into four categories based on their diameter. **(C)** LC3, SQSTM1/p62, and Beclin-1 with matched β-actin blots. Quantification of **(D)** LC3-II, **(E)** LC3-I protein levels and **(F)** its ratio. **(G)** Quantification of LC3 A and **(H)** LC3 B mRNA levels. **(J)** Quantification of SQSTM1/p62 and **(K)** its mRNA level. **(L)** Quantification of Beclin-1 level. The data is presented as a mean ± standard deviation, * indicates statistically significant changes (*p* < 0.05). SD marks a group of control animals fed with standard chow, while experimental mice were maintained on the ketogenic diet composed of fat of animal (KA) or plant (KP) origins.

Analysis of the LC3 puncta by immunofluorescence microscopy (Figure 1A) revealed higher numbers of LC3 puncta in the hippocampi of animals fed with the diet composed of plant-based fat [one-way ANOVA, F(2, 11) = 5.38, p = 0.023, Figure 1B]. The increase of LC3 puncta in the KA group did not reach statistical significance. The analyzed objects were divided into four categories depending on their diameter (Figure 1B). The number of LC3 puncta was consistently elevated in the KP group for all size categories. Observations made by fluorescence microscopy were confirmed by the western blot of autophagy regulating proteins (Figure 1C). The level of LC3-II (LC3-phosphatidylethanolamine conjugate), a marker of autophagosome formation, increased in the hippocampi of animals fed with both ketogenic diets [F(2, 11) = 12.62, p = 0.0014, Figure 1D]. In addition, the level of LC3-I was slightly elevated in the KA group (Kruskal-Wallis H = 6.031, p = 0.04, Figure 1E). The LCII/LCI ratio was changed (Kruskal Wallis H = 11.57, p < 0.0001, Figure 1F) and significantly elevated only in mice fed with the plant fat-based ketogenic diet. Higher levels of LC3-I in the KA group may result from the reduced conversion of LC3-I to LC3-II or the upregulation of LC3 expression on the transcriptional level. To clarify this, the level of LC3 mRNA with qRT-PCR was measured. Relative quantification revealed the significant increase of LC3 A mRNA [**one-**way A**NOVA**, F(2, 11) = 27.32, p < 0.0001, Figure 1G], but not B isoform (Figure 1H) in both ketogenic groups. This suggests that the upregulation of LC3-I measured by immunoblotting arose from the increased expression of this protein. SQSTM1/p62 links ubiquitinated proteins to the autophagic machinery, thus enabling their degradation. Since the protein is itself degraded in autophagolysosomes, it may be used as a marker of autophagy. Although a decrease of SQSTM1/p62 is expected to accompany an elevation of LC3-II, no differences were observed in the levels of SQSTM1/p62 in animals fed with ketogenic diets for***4*** weeks (one-way ANOVA F = 1.054, p = 0.38, Figure 1J). It has been previously shown that during prolonged starvation, the expression of the substrate SQSTM1/p62 is restored depending on transcriptional upregulation (Sahani et al., 2014). Thus, the authors speculate that treatment with a ketogenic diet enhances the transcription of SQSTM1/p62. To verify this, qRT-PCR analysis was performed, which showed the mRNA levels of SQSTM1/p62 were elevated in the hippocampal samples of animals from both ketogenic groups (one-way ANOVA F = 13.64, *p* = 0.0008; *p* = 0.027 for KA vs. SD and *p* = 0.0006 for KP vs. SD, Figure 1K).

As a third marker of autophagy, the levels of Beclin-1 were measured, one of the key regulators of this process. Although the western blot data did not show significant differences in the level of Beclin-1 (one-way ANOVA *F* = 2.16, *p* = 0.16, Figure 1L), a trend toward an increase was observed in the animals fed with ketogenic diets, especially in the KP group.

#### Cerebrocortical Levels of Autophagy Showed More Subtle Changes in Response to Ketogenic Diets

**FIGURE 2:**
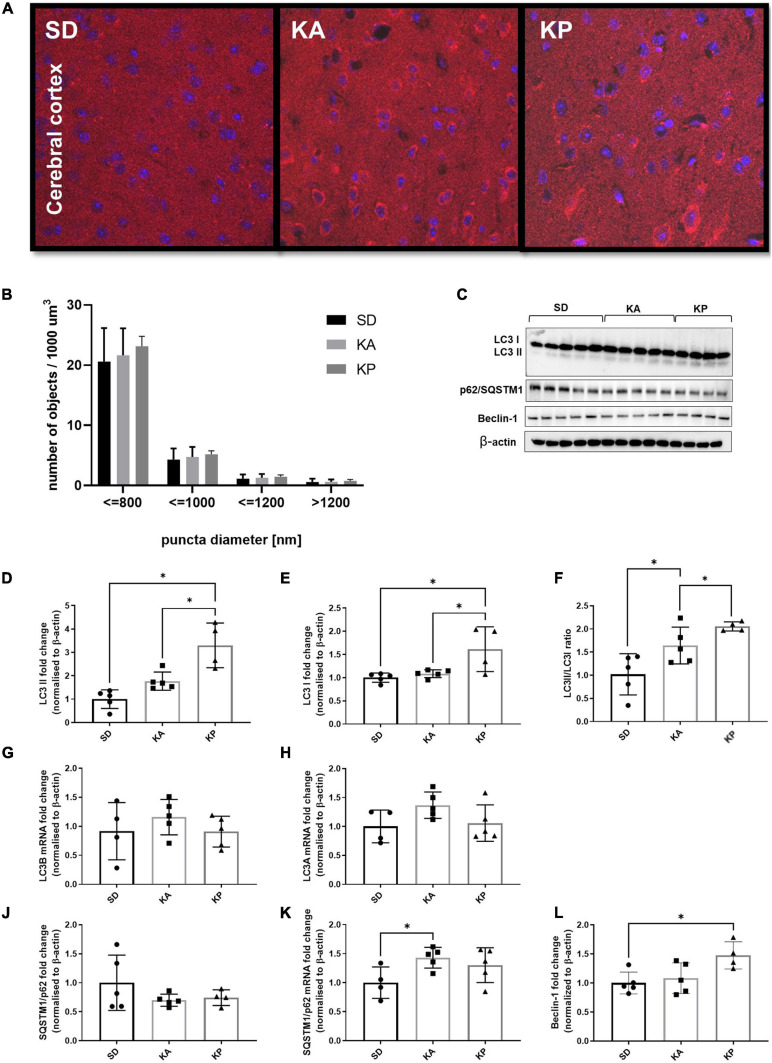
The level of autophagy markers in the cerebral cortex of the mice fed with the ketogenic diets. **(A)** The cortices were immunolabeled against LC3 protein (red) and the level of LC3-positive puncta was counted (*n* = 5). The nuclei are shown in blue (DAPI). **(B)** Number of LC3 puncta per 1 μm^3 of tissue; the counted objects were divided into four categories based on their diameter. **(C)** LC3, SQSTM1/p62, and Beclin-1 with matched β-actin blots. Quantification of **(D)** LC3-II, **(E)** LC3-I protein levels and **(F)** its ratio. **(G)** Quantification of LC3 A and **(H)** LC3 B mRNA levels. **(J)** Quantification of SQSTM1/p62 and **(K)** its mRNA level. **(L)** Quantification of Beclin-1 level. The data is presented as a mean ± standard deviation, ^∗^ indicates statistically significant changes (*p* < 0.05). SD marks a group of control animals fed with standard chow, while experimental mice were maintained on the ketogenic diet composed of fat of animal (KA) or plant (KP) origins.

The analysis of LC3 puncta with confocal microscopy showed trends similar to those observed in the hippocampal samples, although without statistical significance (one-way ANOVA *F* = 0.38, *p* = 0.69; Figures 2A,B). Consistent with the changes observed in the hippocampus, the levels of LC3-II protein in the cerebral cortex were elevated in animals fed with ketogenic diets [one-way ANOVA, *F*(2, 11) = 16.39, *p* = 0.0005; Figures 2C,D]. In the KA group, the LC3-II level was equal to 1.7 times the control levels, but this difference was not statistically significant in the *post hoc* test (*p* = 0.15). However, when comparing cortical LC3-II levels between SD and KA groups with the Student *t*-test, the difference reaches significance (*t* = 3.09, df = 8, *p* = 0.015). The evident increase of LC3-II levels (3.28 times the control levels) was observed in the cerebral cortex of animals fed with the ketogenic diet composed of plant-derived fats (Figure 2D). In the KP group the level of LC3-I was also higher in comparison to both the control and KA group [one-way ANOVA, *F*(2, 11) = 6.8, *p* = 0.012; Figure 2E]. The calculated LC3 II/I ratio was higher in animals fed by both ketogenic diets regardless of the source of fats used [one-way ANOVA, *F*(2, 11) = 9.34, *p* = 0.0043, Figure 2F]. The upregulation of LC3 protein was not reflected by changes in its gene expression, as levels of LC3 mRNA did not differ between animals fed with the ketogenic or standard diets (Figures 2G,H). Similar to the hippocampal samples, the immunoblotting analysis did not reveal significant changes in the level of p62 protein (Figure 2J). The cortical mRNA level of SQSTM1/p62 was significantly elevated in the KA group (Tukey *post hoc* comparison *p* = 0.028), however, the ANOVA analysis did not display any differences among all tested cohorts [one-way ANOVA *F*(2, 11) = 3.29, *p* = 0.076; Figure 2K]. Beclin-1 was upregulated in the KP group in comparison with the control [one-way ANOVA, *F*(2, 11) = 5.23, *p* = 0.025, Figure 2L], which again points to the stronger effect of the plant fat-based ketogenic diet on brain autophagy.

To evaluate the influence of a short-term ketogenic diet on autophagic markers, a second experiment was performed in which animals were fed with ketogenic diets for 24 or 48 h (attached as Supplementary Material). In this experiment, an additional control was introduced—the F24 group of mice food-deprived for 24 h. After 24 h of fasting and ketogenic dieting, the blood BHB concentration increased form 0.45 ± 0.13 in the control to 1.63 ± 0.41, 1.9 ± 0.55, and 1.85 ± 0.31 mM in the F24, KA24, and KP24 groups, respectively. A further increase was observed after the next 24 h of ketogenic diets: to 3.13 ± 0.62 and 4.22 ± 0.66 mM in the KA48 and KP48 groups (data not shown). The immunoblotting analysis of LC3-I, LC3-II, and SQSTM1/p62 did not show any significant differences neither in the hippocampal nor cortical samples (Supplementary Figure 2). The results indicate that 48 h of feeding with a ketogenic diet is not sufficient to induce changes in hippocampal and cerebrocortical autophagy. In the author’s previous study, a 48 h ketogenic diet treatment resulted in a significant increase of LC3-II accompanied by a decrease of SQSTM1/p62 in hepatic samples (Liśkiewicz et al., 2021).

### Both Ketogenic Diets Resulted in the Increased Expression of Ketones Utilizing and Transporting Proteins in the Hippocampus, but Not in the Cerebral Cortex

**FIGURE 3:**
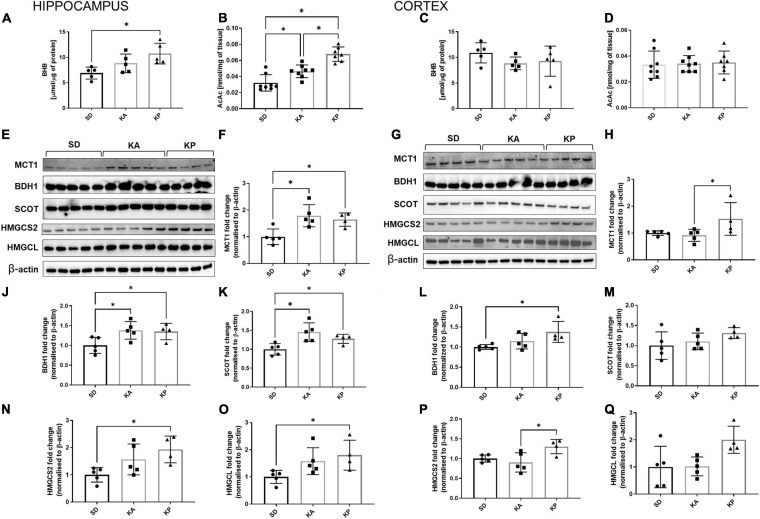
The hippocampal and cortical levels of ketone bodies and enzymes involved in ketone turnover in the cells. The level of hippocampal **(A,B**, *n* = 5) and cortical (**C,D**, *n* = 5) β-hydroksybutyrate (BHB) and acetoacetate levels (AcAc). MCT1, BDH1, SCOT, HMGCS2, and HMGCL matched β-actin blots performed in the hippocampus **(E)** and cortex **(G)**. Quantification of MCT1 levels in the hippocampus **(F)** and cortex **(H)**. Quantification of BDH1 levels in the hippocampus **(J)** and cortex **(L)**. Quantification of SCOT levels in the hippocampus **(K)** and cortex **(M)**. Quantification of HMGCS2 level in the hippocampus **(N)** and cortex **(P)**. Quantification of HMGCL level in the hippocampus **(O)** and cortex **(Q)**. The data is presented as a mean ± standard deviation, ^∗^ indicates statistically significant changes (*p* < 0.05). SD marks a group of control animals fed with standard chow, while experimental mice were maintained on the ketogenic diet composed of fat of animal (KA) or plant (KP) origins.

The levels of ketone bodies BHB and AcAc were measured in the hippocampus and cerebral cortex (Figures 3A–D). In the hippocampus only, the P diet increased the level of BHB [one-way-ANOVA *F*(2, 12) = 6.19, *p* = 0.0142, Figure 3A], while AcAc was elevated by both diets but stronger in the KP group (Kruskal-Wallis H = 16.49, *p* = 0.0003, Figure 3B). BHB and AcAc levels were unchanged in the cortices of the mice fed neither by A nor P diets (Figures 3C,D). To gain some insight into the mechanisms underlying the differences in KB (ketone bodies) levels, the level of MCT1 expression was measured, which is a principal gate of peripheral BHB uptake to the brain. The MCT1 level was higher in the hippocampus of A and P diet-fed mice [one-way-ANOVA, *F*(2, 11) = 7.834, *p* = 0.0077, Figures 3E,F], while in the cortex it was elevated in the mice fed by the P diet (Kruskal-Wallis H = 7.426, *p* = 0.015, Figures 3G,H). As a metabolic substrate, BHB is converted by BDH1 to AcAc and next by SCOT to Acetoacetyl-CoA, leading to the metabolic utilization of ketones. While both ketogenic diets stimulated the expression of hippocampal BDH1 [one-way ANOVA, *F*(2, 11) = 4.872, *p* = 0.031, Figure 3J] and SCOT [one-way ANOVA, *F*(2, 11) = 7.615, *p* = 0.0084, Figure 3K], the ketone utilizing machinery remained unchanged in the cortex (Figures 3L,M) except for the increased level of BDH1 during feeding by the P diet [one-way ANOVA *F*(2, 11) = 4.725, *p* = 0.033, Figure 3L]. BDH1 catalyzes the bidirectional reaction regulation of both ketone utilization and synthesis, thus the levels of two more proteins involved in ketogenesis were measured, namely 3-hydroxy-3-methylglutaryl-COA synthase 2 (HMGCS2) and 3-hydroxymethyl-3-methylglutaryl-CoA lyase (HMGCL). In the hippocampus, the expression of HMGCS2 [one-way ANOVA, *F*(2, 11) = 4.785, *p* = 0.032, Figure 3N] and HMGCL [one-way ANOVA *F*(2, 11) = 4.077, *p* = 0.0473, Figure 3O] were elevated, reaching the highest level in the KP group. In the cortices, similar trends in HMGCS2 [one-way ANOVA, *F*(2, 11) = 5.582, *p* = 0.0212, Figure 3P] and HMGCL [one-way ANOVA, *F*(2, 11) = 4.344, *p* = 0.0407, Figure 3Q] expression were observed.

## Discussion

Here, we present the results of the current study, which aimed to evaluate the impact of nutritional ketosis evoked by ketogenic diets on autophagy and ketone body metabolism in the cerebral cortex and hippocampus.

It is thought that the neuroprotective effect of the ketogenic diet can be largely attributed to the metabolic switch of the brain to burning ketones instead of glucose (LaManna et al., 2009; Zhang et al., 2013). However, in a recent study Hui et al. (2020) performed a quantitative analysis of the labeled circulating metabolites, concluding that during the ketogenic diet the brain mostly utilizes glucose and lactate. This conclusion contradicts the common opinion that KB are a primarily energetic source for neuronal cells during the ketogenic diet (Zhang et al., 2013; Zilberter and Zilberter, 2020), weakening the importance of KB as fuel for the brain. However, Hui et al. (2020) measured metabolites in the whole brain homogenates, leaving unanswered the hypothesis that various brain structures may have different predispositions toward the utilization of ketones. Thus, we decided to raise this question in the current study, showing that metabolic responses to ketogenic diets were distinct in the hippocampus compared to the cerebral cortex. In the hippocampus, the levels of both ketone bodies BHB and AcAc were elevated, which was accompanied by an increase in the levels of ketone transporter proteins and utilization enzymes. In the cerebral cortex, only subtle changes were observed. We reported similar observations earlier showing that these brain structures respond differently to physical activity, suggesting differences in their metabolic profiles (Liśkiewicz et al., 2020). In comparison with the cerebral cortex, the hippocampus is better adapted to efficient fatty acid metabolism, possessing the highest mitochondrial spare respiratory capacity among the brain structures (Liśkiewicz et al., 2020). Therefore, distinct metabolic adaptations are likely to be launched in the hippocampus and the cerebral cortex. Intriguingly, this also applies to the autophagic response. Our results show a strong autophagic response—a large number of LC3 puncta as well as a high level of LC3-II in the hippocampus, while these parameters obviously remained unchanged in the cerebral cortex. This is consistent with previous results showing the different autophagic response of the hippocampus and cerebral cortex as a response to oxygen and glucose deprivation assessed in an *ex vivo* rat brain slice model (Pérez-Rodríguez et al., 2015). Another study demonstrated the differences in the dynamics of cerebrocortical and hippocampal autophagy in response to severe hypoglycemia and the hypoglycemic coma as well as D-BHB administration (Torres-Esquivel et al., 2020). This is particularly interesting in the context of the present results because this study showed that exogenous BHB can modulate autophagy favoring the autophagic flux (Torres-Esquivel et al., 2020).

To understand the mechanisms underlying regional differences in autophagic response, there is a need to investigate the pathways responsible for autophagy activation under nutritional ketosis. Despite many potential interconnections, this relationship remains largely unexplored. To our best knowledge, only one study has shown data to date on the effect of the ketogenic diet on hippocampal autophagy (Wang et al., 2018), and two studies, including our recent results, on liver autophagy (Ochaba et al., 2019; Liśkiewicz et al., 2021).

We observed an increased number of LC3 puncta and an increase in the level of LC3-II form, which indicates the enhanced formation of autophagosomes, after 4 weeks of feeding with ketogenic diets.

No differences in autophagic markers were detected in the investigated brain structures after a short duration of feeding—for 24 or 48 h—while 48 h of feeding with a ketogenic diet was sufficient to induce hepatic autophagy, as we reported previously (Liśkiewicz et al., 2021). This supports the common opinion about the lower levels of basal autophagy and weaker responsiveness of autophagic machinery in the brain in comparison with the peripheral tissues. Intriguingly, in contrast to the liver, we did not observe a reduction in p62 protein levels in the brain of ketogenic diet-fed animals, but we observed that SQSTM1/p62 was upregulated on the transcriptional level. This is in line with a previous study showing that the expression of the autophagy substrate SQSTM1/p62 is restored to basal levels during prolonged starvation depending on transcriptional upregulation (Sahani et al., 2014). The restoration of SQSTM1/p62 was observed in mouse embryonic fibroblasts and HepG2 cells, but not in HeLa and HEK293 cells (Sahani et al., 2014). Together with our results this suggests a tissue-specific effect of the adaptive response of SQSTM1/p62 overexpression to prolonged autophagy activation. However, it should be mentioned here that the stable level of SQSTM1/p62 might be a consequence of reduced autophagic flux and inefficient degradation of autophagic cargo during nutritional ketosis. We would like to emphasize that the measurements performed in the present study allow the reporting of changes in autophagic markers, but are insufficient for determining if the observed changes result from enhanced autophagy or insufficient autophagic flux, which may be considered as a limitation of the present study.

In both brain regions we observed the stronger upregulation of autophagic markers in animals fed with a plant fat-based ketogenic diet, which is consistent with our previous observations regarding hepatic autophagy. We propose that the strength of autophagy enhancement results from the combined effect of nutritional ketosis and the consumption of nutritional factors, which may act as autophagy modulators themselves or contribute to the production of pro- or anti-autophagic metabolites. Interestingly, the changes in the ketone body utilizing and transporting proteins also tend to be stronger after treatment with the plant fat-based ketogenic diet. Why does the plant-based ketogenic chow launch more robust metabolic adaptations? First of all, the diets consist of different compounds which may act as autophagy modulators. The differences most of all concern the fatty acid composition of the chows, inducing changes in the tissue fatty acid content, which we reported in detail previously (Liśkiewicz et al., 2021). As an example, the plant-based diet delivers a high amount of caprylic acid (Liśkiewicz et al., 2021), which is often referred to as the “most ketogenic MCT (Medium Chain Triglycerides)” due to its rapid breakdown from an 8-carbon fatty acid to ketone bodies (Vandenberghe et al., 2017). Secondly, we observe slightly higher levels of blood ketone level in the plant fat-based diet in comparison to the animal fat-based diet in our experiments (although no significant differences were noted in the measurements performed in this experiment).

Finally, the results obtained in the present study open further directions of research—especially regarding the very interesting relationship between local ketone body metabolism and autophagy. The hippocampus showed a stronger response to nutritional ketosis in both changes in the markers of autophagy as well as levels of ketone body utilizing and transporting proteins, which was also accompanied by increased concentrations of ketone bodies in this brain structure. These effects can be unrelated, but in light of the growing body of evidence showing that exogenous BHB administration induces autophagy *in vitro* (Finn and Dice, 2005; Camberos-Luna et al., 2016) and improves autophagic flux in brain cells *in vivo* (Torres-Esquivel et al., 2020), it can be hypothesized that the level of autophagy depends on the local ketone body concentration. On the other hand, it has been shown that autophagy is essential for hepatic and renal ketogenesis during starvation, therefore we cannot exclude the possibility that the relationship between these processes is opposite. The possibility of the existence of extrahepatic ketogenesis in the CNS has been recently raised (Puchalska and Crawford, 2017). It was demonstrated that astrocytes are capable of synthesizing ketones (Le Foll et al., 2014; Non-aka et al., 2016) especially in response to caprylic acid (Thevenet et al., 2016). HMGCS2, HMGCL, and BDH1 are crucial enzymes for ketogenesis. It should be mentioned here that there is inconsistent data regarding HMGCS2 expression in the nervous tissue. While some authors showed a lack of expression of HMGCS2 in astrocytes and neurons (Thevenet et al., 2016), the latest studies confirm the astrocytic expression of HMGCS mRNA (Song et al., 2021). HMGCS has been found to be abundantly expressed in the astrocytes of the optic nerve (Metwally et al., 2019) and other glial cells (Wang et al., 2016) and various brain areas (Shao et al., 2019; Kondo et al., 2020), including the hippocampus (Wang et al., 2020). Moreover, HMGCS2 has been defined as an autophagy regulator (Hu et al., 2017), which raises the role of ketone metabolism as an autophagy enhancer. We observed that the level of the ketogenic proteins was elevated in the hippocampus especially in plant-fat enriched ketogenic diets. The hippocampus is more enriched with astrocytes than the cerebral cortex (Keller et al., 2018), which may contribute to the stronger response of the hippocampus to nutritional ketosis. Taken together, these observations urge us to make a bold hypothesis: ***That*** brain ketogenesis can be launched during a ketogenic diet *in vivo*. Further studies including *in vitro* neural and glial cells as well as organotypic cultures of different brain areas would bring more precise data allowing to mechanistically establish the existence of the brain ketogenesis as well as the dependency between local ketone body metabolism and autophagy. The lack of mechanistic experiments, without which we remain far away from making a final statement on the occurrence of these phenomena under nutritional ketosis, may be considered a limitation of the present study.

## Data Availability Statement

The raw data supporting the conclusions of this article will be made available by the authors, without undue reservation.

## Ethics Statement

The animal study was reviewed and approved by the Local Committee for Animal Experiments in Katowice decision no. 42/2017.

## Author Contributions

DL: conceptualization, methodology, investigation (animal experiments, material collection, western blot), writing original draft, project administration, and funding acquisition. AL: methodology, investigation (animal experiments, material collection, microscopy), and writing original draft. MN-C: investigation, manuscript preparation review and editing (material collection, western blot). MG: investigation (western blot). NP: investigation (western blot), manuscript preparation review and editing. KG: investigation (western blot, qRT-PCR). SS: investigation (microscopy). JB: resources, supervision, funding acquisition, and manuscript preparation review. AM: resources, supervision, funding acquisition, and manuscript preparation review. All authors contributed to the article and approved the submitted version.

## Conflict of Interest

The authors declare that the research was conducted in the absence of any commercial or financial relationships that could be construed as a potential conflict of interest.

## Publisher’s Note

All claims expressed in this article are solely those of the authors and do not necessarily represent those of their affiliated organizations, or those of the publisher, the editors and the reviewers. Any product that may be evaluated in this article, or claim that may be made by its manufacturer, is not guaranteed or endorsed by the publisher.

## Abbreviations

AcAc: acetoacetate
BBB: blood-brain barrier
BDH1: 3-hydroxybutyrate dehydrogenase 1
BHB: β-hydroxybutyrate
CNS: central nervous system
FA: fatty acids
HMGCL: 3-hydroxymethyl-3-methylglutaryl-CoA lyase
HMGCS2: 3-hydroxy-3-methylglutaryl-COA synthase 2
KB: ketone bodies
LC3: microtubule-associated protein 1 light chain 3
MCT: Medium Chain Triglycerides
MCT1: monocarboxylate transporter 1
SCOT/OXCT1: CoA:3-oxoacid CoA transferase
SQSTM1/p62: sequestosome 1.
