# Supplementary material for: Differential Response of Hippocampal and Cerebrocortical Autophagy and Ketone Body Metabolism to the Ketogenic Diet
Source: Front Cell Neurosci. 2021 Aug 11;15:733607. doi: 10.3389/fncel.2021.733607 (PMC8385303; doi:10.3389/fncel.2021.733607)
Supplement: Supplementary file 1 [file Data_Sheet_1.PDF]

frontiers

Supplementary Material

Differential response of hippocampal and cerebrocortical autophagy and ketone body metabolism to ketogenic diet

Liśkiewicz D.[1*], Liśkiewicz A.[2], Nowacka-Chmielewska M.M.[1], Grabowski M.[2,3], Pondel N.[1], Grabowska K.[2,3] , Student S.[4], Barski J.J. [2,3] and Małecki A.[1]

[1] Laboratory of Molecular Biology, Institute of Physiotherapy and Health Sciences, The Jerzy Kukuczka Academy of Physical Education, Katowice, Poland

[2] Department of Physiology, Faculty of Medical Sciences in Katowice, Medical University of Silesia, Katowice, Poland

[3] Department for Experimental Medicine, Faculty of Medical Sciences in Katowice, Medical University of Silesia, Katowice, Poland

[4]Institute of Automatic Control, Silesian University of Technology, Gliwice 44-100, Poland; Biotechnology Centre, Silesian University of Technology, Gliwice 44-100, Poland.

***Corresponding author:**

Daniela Liśkiewicz

The Jerzy Kukuczka Academy of Physical Education

Ul. Mikolowska 72A, 40-065 Katowice

daniela.liskiewicz@gmail.com

Table S1. Composition of the ketogenic and standard chows.

Ketogenic chows<br>**Ingredient [g/100g of chow]**	KA	KP	**Ingredient [g/100g of chow]**	Standard chow SD
Lard	41.46	-	Wheat bran	20
Butter	18	-	Wheat	36.4
Vegetable Shortening (Planta)	-	59.46	Corn	20
Corn oil	9	9	Dried whey	3
Arbocel	9	9	Soybean meal	17
Casein	17.5	17.5		-
Fooder salt	0.4	0.4		0.3
Choline chloride 50%	0.38	0.38		-
Monocalcium phosphate	2	2		0.8
Fodder chalk	1	1		15
Vitamin mix	1	1		1
Methionine	0.26	0.26		-

Table S2. Nutritional profile of the ketogenic and standard chows.

Nutritional Value [g/100g of chow]	Ketogenic chow		Standard chow
	KA	KP	SD
Carbohydrate	1.6	1.6	52.5
Fiber	0.5	0.5	14.3
Protein	12.9	12.7	16.7
Total fat	61.5	59.6	2.1
Ratio (fat:carb+protein)	4.2:1	4.2:1	0.3:1

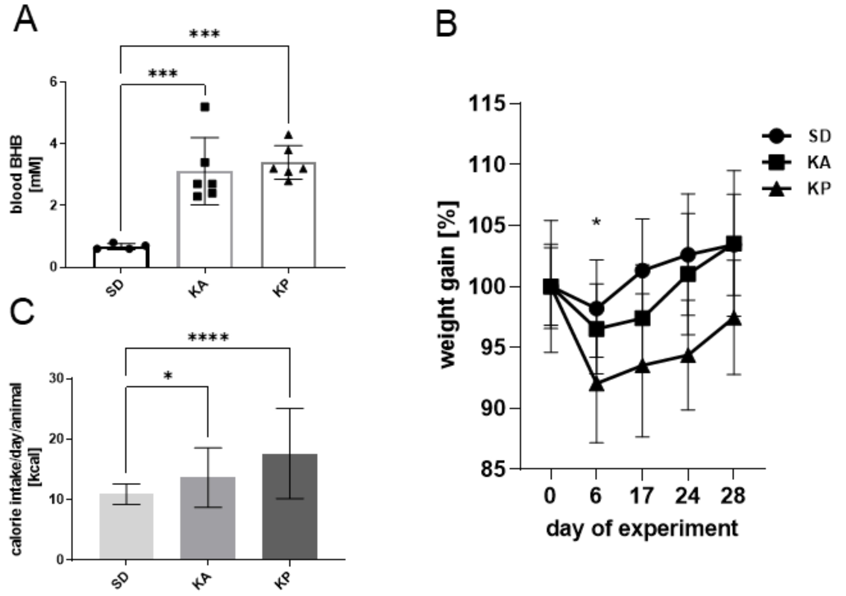

Figure S1 Blood BHB level, animal weight and calorie intake (**A-C**). In the experiment the mice were fed for 4 weeks with ketogenic diets composed of fat of an animal- (KA) or plant- (KP) origin or standard chow (SD). (**A**) Blood β-hydroxybutyrate levels (**B**) Animal weight change is shown as a percentage of the baseline; two-way ANOVA followed by Tukey's test was used. (**C**) Average calorie intake during the course of experiment calculated per day per animal. Data is presented as mean and S.D. * $P < .05$, *** $P < .001$, **** $P < .0001$.

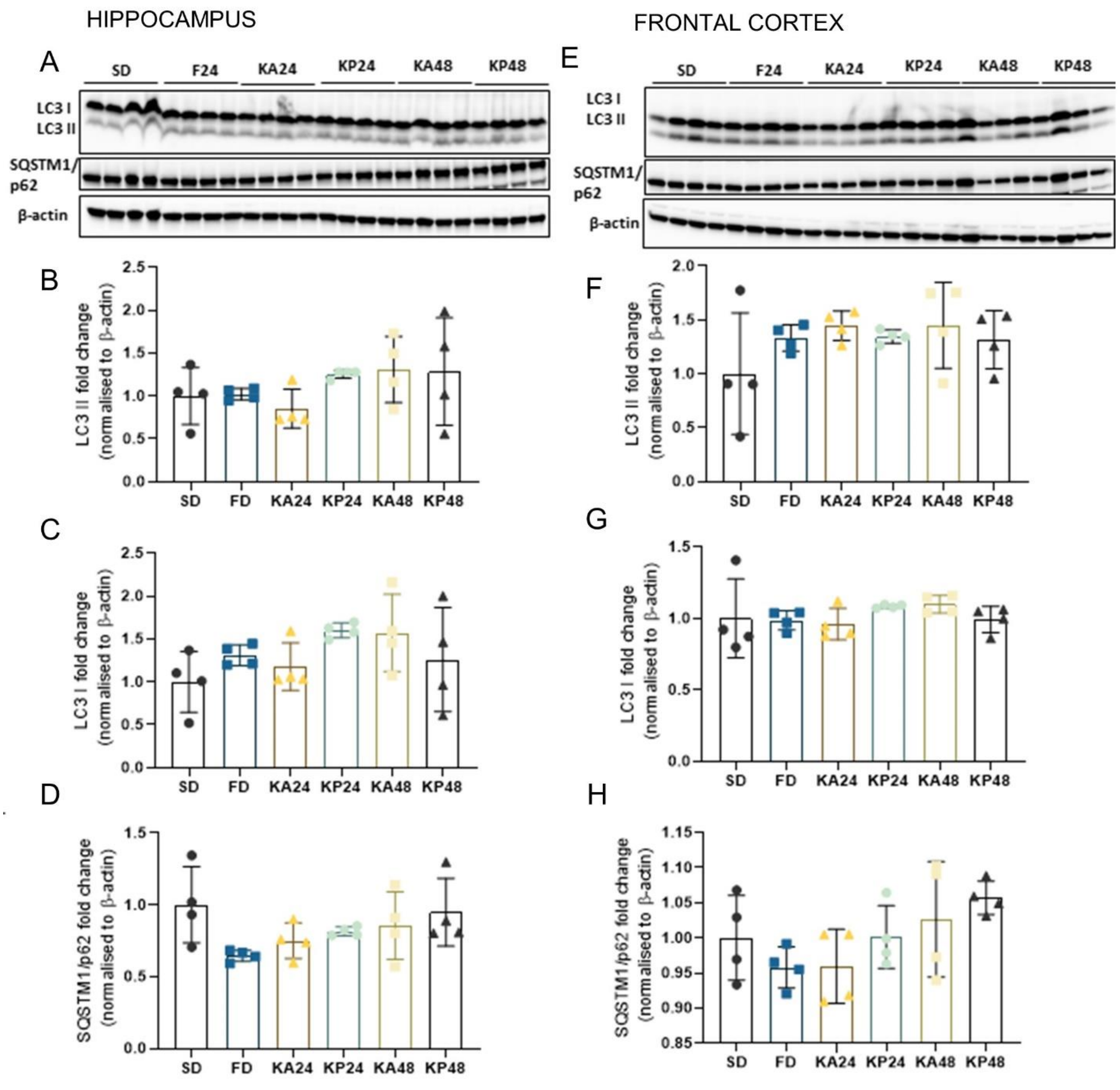

Figure S2. Influence of short-term treatment with ketogenic diets on brain autophagy. (A) In short term experiment, the mice were food deprived or fed with standard chow (SD) or the ketogenic diet composed of fat of an animal or plant origin for 24 (F24, KA24, KP24 groups, respectively) or 48 h (F48, KA48, KP48 groups, respectively). (A) Hippocampal LC3, SQSTM1/p62 and β-actin blots after 24 h or 48 h treatment. Quantification of the hippocampal: (B) LC3-II and (C) LC3-I and (D) SQSTM1/p62 levels. (E) Cortical LC3, SQSTM1/p62 and β-actin blots after 24 h or 48 h treatment. Quantification of the cortical: (F) LC3-II and (G) LC3-I and (H) SQSTM1/p62 levels. One-way ANOVA followed by Tukey's test was used; mean values ± SD are shown on each graph (n = 4 in each group).
